# Berberine improves glucogenesis and lipid metabolism in nonalcoholic fatty liver disease

**DOI:** 10.1186/s12902-017-0165-7

**Published:** 2017-02-28

**Authors:** Li Zhao, Zhen Cang, Honglin Sun, Xiaomin Nie, Ningjian Wang, Yingli Lu

**Affiliations:** Institute and Department of Endocrinology and Metabolism, Shanghai Ninth People’s Hospital Affiliated to Shanghai JiaoTong University School of Medicine, Shanghai, 200011 China

**Keywords:** Nonalcoholic fatty liver disease, Berberine, Glucose and lipid metabolism, Isotope tracer

## Abstract

**Background:**

Nonalcoholic fatty liver disease (NAFLD) is considered a critical hepatic manifestation of metabolic syndrome. Berberine (BBR) exerts anti-hyperglycemic and anti-dyslipidemic effects and can also ameliorate NAFLD. Thus, BBR might exert its therapeutic effect on NAFLD by improving glucolipid metabolism. Here, we investigated the aspects and extent to which glucolipid metabolism were affected by BBR in rats with NAFLD.

**Methods:**

Three groups of Sprague–Dawley rats were studied: a control group (*n* = 6) fed a normal chow diet and a NAFLD group (*n* = 6) and a NAFLD + BBR group (*n* = 6) fed a high-fat diet. Normal saline and BBR (150 mg/kg body weight/day for 16 weeks) were administered by gavage. All rats were infused with isotope tracers. The rates of glucose appearance (Ra_glu_), gluconeogenesis (GNG) and glycerol appearance (Ra_gly_) were assessed with ^2^H and ^13^C tracers, whereas the rates of hepatic lipogenesis and fatty acid β oxidation were measured using the ^3^H tracer.

**Results:**

When the NAFLD model was successfully induced by administering a high-fat diet, body weight, insulin resistance and dyslipidemia were significantly increased. After the BBR treatment, weight loss, decreased lipid profiles and HOMA-IR, and increased ISI were observed. Meanwhile, BBR reduced Ra_glu_, GNG and hepatic lipogenesis, whereas the rate of fatty acid β oxidation in skeletal muscle showed an increasing trend. Ra_gly_ showed a decreasing trend. Based on the results of the histological analysis, BBR obviously attenuated the ectopic liver fat accumulation.

**Conclusions:**

BBR improved NAFLD by inhibiting glucogenesis and comprehensively regulating lipid metabolism, and its effect on inhibiting hepatic lipogenesis was much stronger. The improvement may be partly mediated by weight loss. Berberine might be a good choice for patients with NAFLD and glucose metabolic disorder. Future clinical trials need to be conducted to confirm these effects.

## Background

NAFLD has become the second most common liver disease in China after viral hepatitis [1]. Due to the alterations in lifestyle and the epidemic of obesity, the prevalence of NAFLD is increasing worldwide [2]: up to 30% in developed countries and nearly 10% in developing nations [3]. NAFLD encompasses the whole spectrum of liver diseases that are not associated with significant alcohol consumption, including simple hepatic steatosis, non-alcoholic steatohepatitis (NASH), cirrhosis, liver failure and even hepatocellular carcinoma (HCC) [4]. However, the precise mechanisms underlying the pathogenesis of NAFLD remain unclear. A well-known “two-hit” hypothesis has been proposed for NAFLD progression [5]. The “first hit” is the development of hepatic steatosis as a result of insulin resistance, whereas in the “second hit”, proinflammatory mediators induce hepatic inflammation, hepatocellular injury, fibrosis and cirrhosis. Furthermore, lifestyle modifications are the only suggested remedy to avoid progression of benign steatosis to NASH and fibrosis [6]. With the exception of preventive lifestyle intervention, anti-diabetic, lipid-lowering or anti-hypertensive agents may be used to control NAFLD comorbidities. Currently, no approved pharmacological agents are available for NAFLD [5]. Recently, Chinese herbs, including berberine (BBR), have received more attention as treatments for NAFLD.

BBR, an isoquinoline alkaloid, is a natural compound in numerous Chinese herb plants such as *Berberisaristata*, *Coptischinensis*, *Coptis rhizome*, etc. [7]. Low doses of BBR have been widely used to treat intestinal bacteria-related diarrhea with good safety for thousands of years. Over the last few decades, many animal studies and clinical trials have reported the anti-hyperglycemic and anti-dyslipidemic effects of BBR [8–10]. Interestingly, in these investigations, BBR was also reported to have a potent effect on reducing hepatic steatosis [8, 10]. Although the bioavailability of BBR was reported to be less than 1% in some studies [11, 12], other studies indicated that BBR was typically concentrated in the liver (at levels 50–70 times higher than the plasma levels) after oral administration [10, 13]. Moreover, in some animal experiments, BBR was shown to alleviate TG deposition in the liver following an intraperitoneal injection or oral gavage [6]; therefore, BBR is indeed suitable as a treatment for NAFLD [13]. However, the mechanism underlying its therapeutic effect is still unclear. Many possible metabolic pathways have been suggested, such as decreasing the severity of liver and adipose tissue inflammation [4], modulating ER stress [6], regulating the expression of hepatic genes related to glucolipid metabolism [10] and gut microbiota [14], etc. However, comprehensive studies on the aspects and extent to which glucolipid metabolism in visceral and peripheral tissues in NAFLD were affected by BBR are lacking.

In the present study, we used isotope tracers to explore the effect of BBR on hepatic and extra hepatic glucolipid metabolism in rats with NAFLD.

## Methods

### Animal model establishment and experimental design

Eighteen male Sprague-Dawley rats (8 weeks old) were obtained from Shanghai Laboratory Animal Center in China. The animals were maintained on a 12/12-h light/dark cycle in a temperature-controlled room (22 ± 2 °C) and given free access to food and water. After 1 week of acclimation, the animals were randomly divided into two groups: a control group (NCD group, *n* = 6) receiving a normal chow diet (NCD:65.5% carbohydrate, 20% protein and 10.3% fat) and a HFD group (*n* = 12) fed a high-fat diet (HFD:40% carbohydrate, 20% protein and 40% fat). After 16 weeks, the NAFLD Model was successfully induced in these 12 rats (verified by ultrasound diagnosis), which were further subdivided into two groups (6 rats per group): (i) the NAFLD group, which was administered an equal volume of normal saline, and (ii) the NAFLD + BBR group, which was treated with 150 mg BBR/kg body weight/day (Sigma-Aldrich, USA) by gavage for 16 weeks.

Body weights and fasting blood glucose (FBG) levels were measured every 2 weeks. Glucose levels were immediately measured using an electronic glucometer (Terumo, Tokyo, Japan). Lipid profiles, including the total cholesterol (TC), triglyceride (TG), low-density lipoprotein-cholesterol (LDL) and free fatty acid (FFA) and insulin levels, were assayed at the 0^th^, 16^th^, and 32^nd^ weeks using Siemens Dimension MAX (Siemens Healthcare Diagnostics Inc.) and ELISA kits (Shibayaji, Japan). Then, the HOMA-IR and insulin sensitivity index (ISI) were calculated from the FBG and fasting insulin (FINS) levels using the following detailed formulas: HOMA-IR = FBG*FINS/22.5; ISI = Ln[1/(FBG*FINS)] [15]. All experimental procedures were conducted in accordance with the ethical principles in animal research adopted by the Department of Laboratory Animal Science and approved by the Animal Experimental Ethical Committee of Jiaotong University School of Medicine, Shanghai, China.

### Isotope infusion

**Fig. 1:**
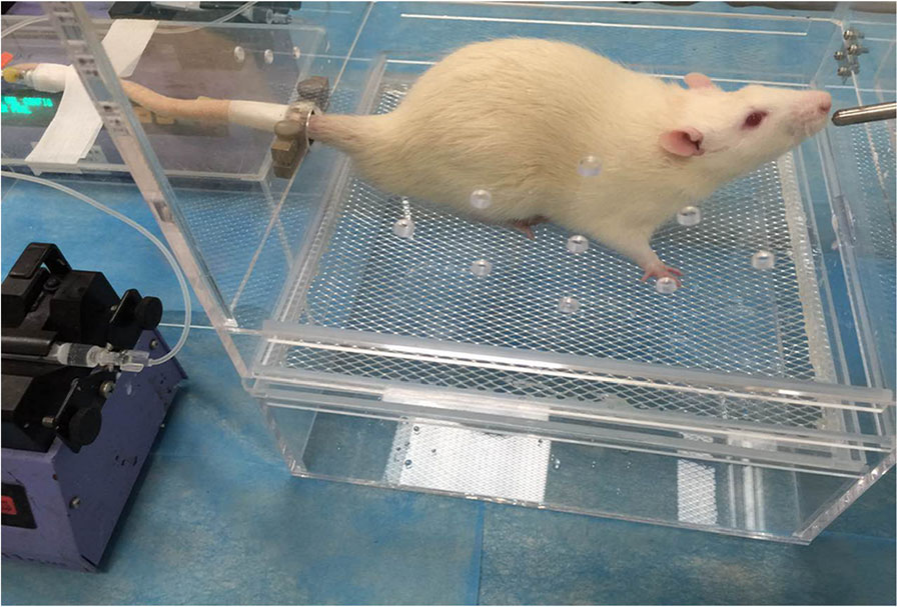
Experimental conditions used for tracer perfusion. The lateral tail vein was catheterized for tracer infusion and the tail artery was catheterized for blood collection. Throughout the experiments, all rats were conscious and relaxed, with the ability to groom themselves normally and drink water freely. Thus, the experimentally induced stress was minimized

**Fig. 2:**
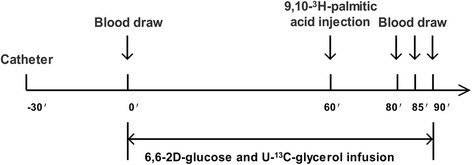
Protocol for the isotope tracer infusion. This figure shows the process used for tracer infusion and blood collection

All rats were fasted overnight and studied the following morning. After local anesthesia was induced with lidocaine, the lateral tail vein was catheterized for the infusion of tracers and the tail artery was catheterized for blood sampling using previously described methods [16]. The animals were conscious and relaxed throughout the experiments; in addition, the animals could groom themselves normally, drink water freely or sit calmly to minimize experimentally induced stress (Fig. 1). When the FBG levels returned to baseline (usually within 30 min), 6,6-2D-glucose (2 μmol/kg/ min) and U-^13^C-glycerol (0.84 μmol/kg/min) were constantly infused through the i.v. infusion line driven by a Harvard mini-infusion pump (Harvard Apparatus, Holliston, MA, USA) for 90 min. At 60 min, 1 μCi of 9,10-^3^H-palmitic acid was injected. During the final 10 min, three arterial blood samples (0.5 ml each) were collected at 5 min intervals and were used to quantify steady state glucose and glycerol metabolism (Fig. 2). Then, the animals were euthanized by opening the heart under anesthesia with pentobarbital (50 mg/kg). A strip of gastrocnemius muscle (approximately 13*3*1 mm) was promptly obtained to examine β oxidation of 9,10-^3^H-palmitic acid (1 μCi) in vitro. Livers were also harvested swiftly, immersed in liquid nitrogen and stored at −80 °C. Plasma samples were prepared on ice, centrifuged at 4 °C, separated and stored at −80 °C until further analysis.

### Measurement of isotope tracers

Plasma samples were processed with methoxyamine-HCl and BSTFA to obtain the trimethylsilyl derivatives of 6, 6-2D-glucose and U-^13^C-glycerol. Hydroxylamine hydrochloride was used for derivation to avoid interference from the reaction of 6,6-2D-glucose to 1,2,3-^13^C-glucose formed in GNG via U-^13^C-glycerol. Then, the enrichment of the derivatives was measured using gas chromatography/mass spectrometry (GC-MS, Agilent 5975C, Agilent Technologies). Ions with mass-to-charge ratios (m/z) of 319 (unlabeled glucose) and 321 (labeled glucose) were monitored. The peak area 321/319 ratio was calculated, and the corresponding enrichment was determined from standard curves. A similar method was used to obtain the m/z 221/218 U-^13^C-glycerol and m/z 215/212 1,2,3-^13^C-glucose ratios and determine their corresponding enrichment.

Lipids were extracted from the liver using the Folch method [17], and triglyceride concentrations were assayed using an ELISA kit (Jiancheng, Nanjing, China). Then, pure triglycerides were isolated using thin layer chromatography (TLC). In addition, ^3^H_2_O converted from 9,10-^3^H-palmitic acid during the process of β oxidation was obtained by removing the lipids with chloroform. ^3^H radioactivity from both liver triglycerides and ^3^H_2_O was determined using liquid scintillation counting (LS6500 Multipurpose Scintillation Counter, Beckman, USA) as previously described [16].

### Calculations

The appearance rates (Ra) of glucose and glycerol were calculated with the steady-state equation from the respective tracer infusion rates (F) and mole percent excess (MPE). The infusion rate of 6,6-2D-glucose was divided by the MPE of plasma glucose to yield Ra_glu_ [18]. The detailed formula for Ra_gly_ was described previously [16]. The glycerol gluconeogenesis rates were calculated using the following formula: 1,2,3-^13^ C-glucose MPE*Ra_glu_ /U-^13^C-glycerol MPE [18]. The percent of glycerol converted to glucose was calculated using the following formula: Ra_gly_*^13^C-glucose MPE/F_gly_. Fatty acid β oxidation rates were deduced by measuring the specific activity of ^3^H_2_O. Moreover, the hepatic fat synthesis rate was calculated by dividing the total concentrations of triglycerides by the radioactivity of the corresponding labeled triglycerides [19].

### Liver pathomorphology

After the rats were sacrificed, part of their livers was fixed in 4% paraformaldehyde, dehydrated with ethanol and xylene, embedded in paraffin, and sliced into 5 μm sections on a microtome (SLEE, Germany). The sections were stained with hematoxylin and eosin (H&E) and analyzed under an optical microscope (CKX41, Olympus, Japan) to examine the pathologic structures of the livers.

### Statistical analysis

The software package SPSS version 17.0 was used for data analysis. All data are presented as the means ± standard deviations (SD) and statistical significance was assessed by one-way ANOVA followed by LSD for multiple comparisons as appropriate. *P* < 0.05 was considered statistically significant.

## Results

### Berberine reduced body weight gain and regulated the FBG levels

**Fig. 3:**
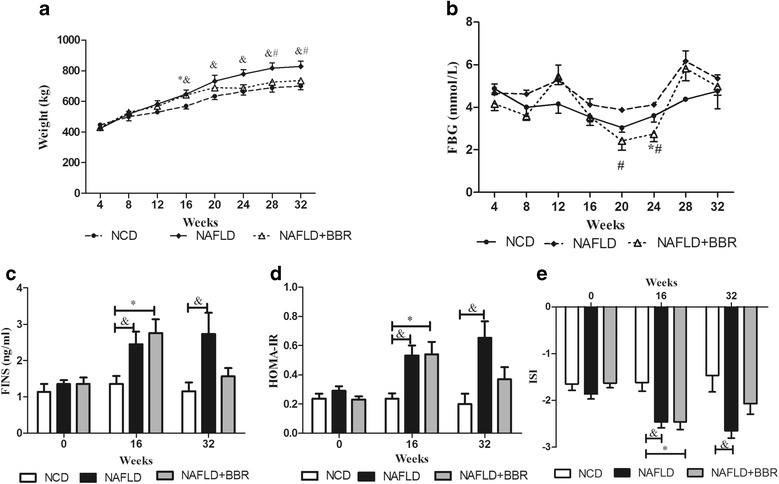
Changes in body weights, FBG levels, FINS levels, HOMA-IR and ISI in the three groups before and after the BBR intervention. **a** A significant increase in body weight was observed at the 16^th^ week after the successful establishment of the NAFLD model. After the 16 week BBR intervention, the NAFLD + BBR group exhibited a significant reduction in body weight compared with NAFLD group.**b** During the first 8-week treatment period, the FBG levels were obviously decreased, but differences were not observed between the three groups at the end of the treatment. After the NAFLD model was successfully established, the FINS concentrations (**c**) and HOMA-IR (**d**) increased significantly and the ISI (**e**) decreasing dramatically. BBR reduced the FINS concentrations, HOMA-IR and increased the ISI at the 32^nd^ week, although the differences were not significant. The data are presented as the means ± SEM. *P < 0.05, NCD vs NAFLD + BBR; &*P* < 0.05, NCD vs NAFLD; #*P* < 0.05, NAFLD vs NAFLD + BBR

Prior to the experiment, no differences were observed in body weights or FBG levels between the three groups. When the NAFLD model was successfully established, a significant increase in body weight was observed (NAFLD: 648 ± 69.95; NAFLD + BBR: 641.67 ± 35.90; NCD: 568.5 ± 36.09, *P* < 0.05), but the FBG levels remained unchanged. After the 16-week BBR intervention, the NAFLD + BBR group (736 ± 20.80) exhibited a significant reduction in body weight compared with the NAFLD group (828.67 ± 86.78) (*P* < 0.05, Fig. 3a). However, the FBG levels were obviously decreased during the first 8-week treatment period (*P* < 0.05, NCD vs NAFLD vs NAFLD + BBR: 3.60 ± 0.59 vs 4.12 ± 0.28 vs 2.74 ± 0.78, respectively), whereas similar FBG levels were observed in the three groups (NCD vs NAFLD vs NAFLD + BBR: 4.77 ± 1.44 vs 5.35 ± 0.42 vs 4.98 ± 0.84, respectively) at the end of the experiment (Fig. 3b).

### Berberine improves insulin resistance

After 16 weeks of feeding the HFD, the FINS concentrations (NAFLD: 2.44 ± 0.87; NAFLD + BBR: 2.75 ± 0.85) and HOMA-IR (NAFLD: 0.53 ± 0.14; NAFLD + BBR: 0.54 ± 0.17) in the two HFD groups were significantly higher than the values in the NCD group (FINS: 1.36 ± 0.48; HOMA-IR: 0.23 ± 0.07, *P* < 0.05). However, no differences were observed between NAFLD group and NAFLD + BBR group (Fig. 3c and d). In contrast, the NCD group (−1.46 ± 0.50) had a higher ISI than the two HFD groups (NAFLD: −2.46 ± 0.25; NAFLD + BBR: −2.46 ± 0.32, *P* < 0.05), but no difference was observed between the latter two groups (Fig. 3e). However, after the 16 weeks intervention with BBR, the NAFLD + BBR group showed an obvious improvement in the FINS concentrations (1.57 ± 0.39), HOMA-IR (0.37 ± 0.15) and ISI (−2.07 ± 0.39) compared with the NAFLD group (2.73 ± 1.18, 0.65 ± 0.22, −2.65 ± 0.31, respectively), although the differences were not statistically significant.

### Berberine attenuated the plasma lipid profiles

**Fig. 4:**
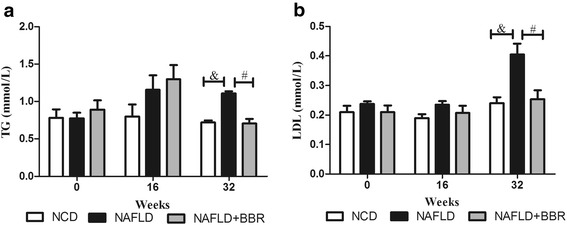
Changes in the TG and LDL concentrations in the three groups at the 0^th^, 16^th^, and 32^nd^ weeks. The TG (**a**) and LDL (**b**) levels were higher in the two HFD groups at the 16^th^ week, but the differences between the groups were not significant. Over the next 16 weeks, BBR reversed the elevated TG and LDL levels to levels similar to the NCD group. The data are expressed as the means ± SEM. &*P* < 0.05, NCD vs NAFLD; #*P* < 0.05, NAFLD vs NAFLD + BBR

Compared to the NCD group (TG: 0.80 ± 0.33; TC: 1.56 ± 0.18; LDL: 0.19 ± 0.02), the two HFD groups had higher TG (NAFLD: 1.16 ± 0.43; NAFLD + BBR: 1.30 ± 0.38), TC (NAFLD: 1.67 ± 0.13; NAFLD + BBR: 1.76 ± 0.17) and LDL (NAFLD: 0.24 ± 0.03; NAFLD + BBR: 0.21 ± 0.06) levels beginning at the 16^th^ week, but the differences between groups were not significant (*P* > 0.05). Over the next 16 weeks, the rats with NAFLD had the highest plasma TG (1.11 ± 0.05), TC (1.92 ± 0.44) and LDL (0.41 ± 0.09) levels. BBR reversed the elevated plasma lipid profiles of the NAFLD + BBR group (TG: 0.70 ± 0.16; TC: 1.55 ± 0.21; LDL: 0.25 ± 0.07) to levels similar to the NCD group (TG: 0.72 ± 0.05; TC: 1.66 ± 0.06; LDL: 0.24 ± 0.03), particularly the TG and LDL levels (Fig. 4a and b). During the entire trial, differences were not observed in the FFA levels between the three groups (*P* > 0.05, data not shown).

### Berberine decreased Ra_glu_, Ra_gly_, GNG from glycerol and the percent of glycerol converted to glucose (%)

**Fig. 5:**
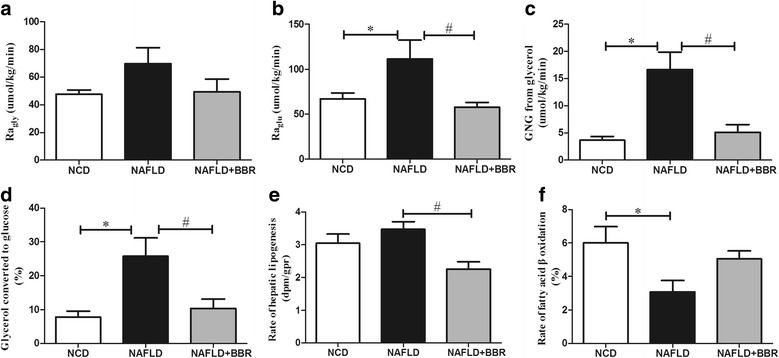
Changes in Ra_gly_, Ra_glu_, GNG from glycerol, the percent of glycerol converted to glucose, rates of hepatic lipogenesis and rates of fatty acid β oxidation in skeletal muscle. Ra_gly_ (**a**) showed a decreasing trend after the BBR treatment. BBR dramatically decreased Ra_glu_ (**b**), GNG from glycerol (**c**) and the percent of glycerol converted to glucose (**d**). In addition, BBR improved hepatic lipogenesis (**e**) and obviously promoted fatty acid β oxidation (**f**). The data are expressed as the means ± SEM. *P < 0.05, NCD vs NAFLD; #*P* < 0.05, NAFLD vs NAFLD + BBR

Although significant differences in Ra_gly_ were not observed between the three groups, Ra_gly_ was still the highest in the NAFLD group (69.74 ± 27.95) and was similar in the NCD (47.64 ± 6.36) and NAFLD + BBR (49.36 ± 18.60) groups (Fig. 5a). Ra_glu_ was significantly increased in the NAFLD group (111.32 ± 51.88, *P* < 0.05, vs NCD or NAFLD + BBR), whereas the NCD (67.24 ± 12.68) and NAFLD + BBR (57.97 ± 10.44) groups had similar Ra_glu_ values (Fig. 5b). The GNG from glycerol primarily increased in the NAFLD group (16.64 ± 7.93, *P* < 0.05, vs NCD or NAFLD + BBR), whereas it was comparable in the NCD (3.63 ± 1.44) and NAFLD + BBR (5.09 ± 2.82) groups (Fig. 5c). Similarly, the percent of glycerol converted to glucose displayed the same trend (*P* < 0.05, NAFLD: 25.82 ± 13.03%; NCD: 7.81 ± 3.49%; NAFLD + BBR: 10.32 ± 5.57%) (Fig. 5d).

### Berberine inhibited hepatic lipogenesis and promoted fatty acid β oxidation in skeletal muscle

The new synthesis of triglycerides in the liver displayed an increasing trend in the NAFLD group (3.47 ± 0.53 vs NCD: 3.05 ± 0.56, *P* > 0.05). Compared with the NAFLD group, BBR significantly reduced hepatic lipogenesis (NAFLD + BBR: 2.25 ± 0.44, *P* < 0.05) (Fig. 5e). The rates of fatty acid β oxidation in skeletal muscle decreased dramatically in the NAFLD group (3.07 ± 1.70%, *P* < 0.05), but no difference was observed between the NCD (6.01 ± 1.93%) and NAFLD + BBR (5.04 ± 0.98%) groups (Fig. 5f).

### Changes in liver morphology

**Fig. 6:**
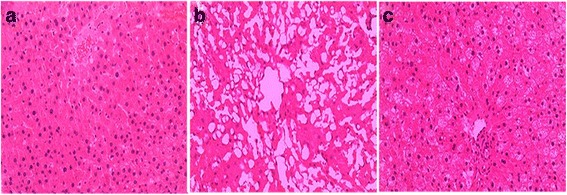
Histopathological changes in the liver (H&E staining, magnification × 200). Compared with the NCD group (**a**), the NAFLD group (**b**) exhibited a disordered arrangement of the hepatic lobules and fatty degeneration of hepatocytes. BBR noticeably improved the injury to the hepatic lobules and hepatocyte steatosis in the rats in the NAFLD + BBR group (**c**)

Under the light microscope, normal liver tissue structures and well-arranged hepatic lobules without liquid droplets were observed in the NCD group (Fig. 6a), whereas a disordered arrangement of the hepatic lobules and fatty degeneration of the hepatocytes was observed in the NAFLD group (Fig. 6b). However, the injury to the hepatic lobules and hepatocyte steatosis observed in the rats in the NAFLD + BBR group were all noticeably improved (Fig. 6c).

## Discussion

NAFLD is already considered a critical hepatic manifestation of metabolic syndrome [4]. In addition, dietary habits and genetic background are thought to be responsible for the pathogenesis and development of hyperlipidemia with NAFLD; therefore, many mouse and rat models of NAFLD have been induced by feeding the animals a high-fat-diet in previous studies [4, 20, 21]. In our study, the rat model of NAFLD was also successfully established by providing nourishment with a high-fat diet for 16 weeks, at which time, the body weight was greatly increased. Meanwhile, increased fasting insulin concentrations, HOMA-IR and decreased ISI were observed in the rats with NAFLD. This result verified that obesity and insulin resistance are two main risk factors for the initiation or exacerbation of NAFLD [14]. After the BBR treatment, obvious weight loss was observed. The possible mechanism was associated with changes in the expression of multiple key genes controlling energy expenditure [22]. Previous studies found that at least a 5–7% of weight loss was required to improve hepatic steatosis [23]. Weight loss could improve hepatic insulin resistance and attenuate liver fat accumulation by reducing FFA flux to the liver for hepatic de novo lipogenesis [24]. Also, weight loss likely attenuates mitochondrial oxidative flux by alleviating the load of FFA and lipotoxicity to hepatic mitochondria [25]. Thus, weight loss is one of the cornerstones of treatment of NAFLD. Moreover, the fasting insulin levels and HOMA-IR decreased, and ISI obviously increased, although the differences were not significant. However, in some previous animal experiments, BBR significantly attenuated insulin resistance [4, 6]. This finding may be associated with the different species of animals and the different degree of obesity induced by the HFD. In addition, concomitant reductions in the total cholesterol, triglyceride, and low-density lipoprotein-cholesterol levels were observed in the BBR-treated rats, and the levels of the latter two were dramatically reduced. These findings may be attributed to the observation that berberine modulates the gut microbiota by up-regulating intestinal Bacteroidetes-to-Firmicutes ratio, which might decrease the animals’ capacity to harvest energy from the diet. Moreover, berberine increases the levels of serum glucagon-like peptide-1 and neuropeptide Y and decreases the levels of orexin-A, which may also modify the gut bacteria and regulate food intake, energy metabolism, circadian rhythm, etc. [26]. Therefore, based on these results, BBR indeed exerted a certain protective effect against hepatic steatosis.

According to the results of the isotope perfusion analysis, Ra_gly_ was much lower in the NAFLD + BBR group than that in the NAFLD group, although the differences between the three groups were not significant. Glycerol is one of the products of lipolysis. During fasting, the main source of glycerol is peripheral adipose tissue degradation. Moreover, glycerol released by lipolysis cannot be resynthesized into adipose tissue, since it lacks glycerokinase. Therefore, the Ra_gly_ in the blood can reflect the extent of lipolysis [16]. According to the results, BBR partially inhibited lipolysis in the rats with NAFLD. In fact, the obesity-associated dysfunction of adipose tissue plays an important role in the development of NAFLD, since adipose tissue not only delivers excess free fatty acids to the liver to facilitate hepatic steatosis but also secretes proinflammatory factors to trigger or exacerbate liver inflammation [4]. Although the levels of the associated inflammatory factors were not detected in the current study, BBR, a recognized anti-inflammatory drug, certainly had a powerful anti-inflammatory effect on both hepatocytes and adipocytes.

We also used 9,10-^3^H-palmitic acid to assess hepatic lipogenesis in vivo and its metabolic utilization in skeletal muscle in vitro. 9,10-^3^H-palmitic acid has many of the same features as normal fatty acids; it is able to be taken up by the liver for triglyceride synthesis and by skeletal muscle to produce ^3^H_2_O via β oxidation. The results showed that BBR significantly reduced the rates of hepatic lipogenesis, whereas the BBR treatment increased the rates of fatty acid β oxidation in skeletal muscle. In addition, liver histology revealed that BBR obviously attenuated ectopic fat accumulation in the liver, consistent with previous findings showing that oral administration of BBR alleviates TG deposition in the liver [6]. Based on these results, BBR comprehensively improved lipid metabolism in NAFLD by inhibiting hepatic lipogenesis and lipolysis in adipose tissue, as well as by promoting fatty acid β oxidation in skeletal muscle. Moreover, BBR exerted the strongest effects on hepatic lipogenesis and fat deposition, possibly because BBR was typically concentrated in the liver (at levels 50–70 times higher than the plasma levels) after oral administration [10, 13]. BBR may have improved lipolysis in adipose tissue and fatty acid β oxidation in skeletal muscle by adjusting energy metabolism pathways, such as PPAR signaling pathways; however, additional animal experiments are required to confirm this hypothesis.

In the basal state, hepatic glucose production (HGP) is equivalent to the glucose appearance rates (Ra_glu_) after an overnight fast. In the current study, the highest Ra_glu_ was observed in NAFLD group and was apparently reduced in the BBR-treated rats. The FBG levels were also significantly decreased in the first 8 weeks of BBR treatment, although there were no differences between the three groups at the end of the experiment. Gluconeogenesis is one of the major mechanisms used to maintain normal FBG levels [16]. Gluconeogenesis was primarily increased in rats with NAFLD and significantly decreased (including glycerol converted to glucose (%) and GNG from glycerol) in the BBR-treated rats compared with the vehicle-treated rats in this study, indicating that BBR attenuated high-fat-diet-induced GNG from glycerol. As described above, insulin resistance, with an increased HOMA-IR and decreased ISI, was apparent in the NAFLD group, but the FBG levels remained within the normal range. Thus, the FBG levels alone cannot predict the metabolic risk in NAFLD. However, the increased Ra_glu_ and GNG in combination with the unaffected FBG levels indicate that the dynamics of glucose metabolism have been actively initiated in the early state. BBR may not only have a beneficial anti-hyperglycemic effect but may also begin to decrease hepatic glucose production in the early stage of glucose metabolic disorder, suggesting that BBR may be a more effective therapeutic strategy for patients with NAFLD and glucose metabolic disorder. Based on these findings, BBR might be an effective treatment to prevent the progression of prediabetes to diabetes, but further animal studies and human trials are required to confirm this hypothesis.

In our study, we comprehensively observed the effects of BBR on hepatic and extra hepatic glucose and lipid metabolism in rats with NAFLD using isotope tracer technology. Tracer techniques have been widely used to study the metabolism of glucose, lipid and other molecules. Previous tracer studies in rat models often involved invasive surgical placement of catheters in the carotid artery and jugular vein [27], which would obviously cause stress. This stress may considerably interfere with the metabolic flux of the substrate and thus affect the results. In the present study, we inserted catheters into the rats’ tail arteries and veins and maintained the rats in a conscious and relaxed state throughout the experiment to limit and reduce stress and to prevent disturbances in glucose and lipid metabolism (Fig. 1). However, our study has some limitations. For example, a single dose of BBR was used; therefore, we could not observe the effects of different doses of BBR on NAFLD. In future studies, we will make up for these deficiencies and further explore the molecular biological mechanism by which BBR regulates glucose and lipid metabolism.

## Conclusions

BBR, a compound derived from herbal medicine, improved NAFLD and prevented its metabolic disorder-related complications by comprehensively regulating glucose and lipid metabolism. The improvement may be partly mediated by weight loss. The most important and beneficial effects of BBR on NAFLD were to inhibit hepatic lipogenesis and fat deposition. In clinical practice, BBR may have a better therapeutic effect on patients with NAFLD and glucose metabolic disorder. Further clinical trials need to be conducted to confirm these effects.

## Abbreviations

BBR: Berberine
FBG: Fasting blood glucose
FFA: Free fatty acid
FINS: Fasting insulin
GNG: Gluconeogenesis
HOMA-IR: Insulin resistance index
ISI: Insulin sensitivity index
LDL: Low-density lipoprotein
NAFLD: Nonalcoholic fatty liver disease
Ra_glu_: Rate of glucose appearance
Ra_gly_: Rate of glycerol appearance
TC: Total cholesterol
TG: Total triglycerides
